# Clinical and Radiographic Features of Mandibular Third Molar Gemination: A Case Report and Literature Review

**DOI:** 10.1155/crid/8934034

**Published:** 2025-05-20

**Authors:** Matteo Pellegrini, Giorgia Creminelli, Pierluigi Guerrieri, Andrea Scribante, Danilo Fraticelli, Luca Creminelli

**Affiliations:** ^1^Maxillofacial Surgery and Dental Unit, Fondazione IRCCS Cà Granda Ospedale Maggiore Policlinico, Milan, Italy; ^2^Department of Biomedical, Surgical and Dental Sciences, University of Milan, Milan, Italy; ^3^Section of Dentistry, Department of Clinical, Surgical, Diagnostic and Pediatric Sciences, University of Pavia, Pavia, Italy; ^4^Private Practitioner, Villanterio, Italy

**Keywords:** dental anomalies, dental gemination, dentistry, double tooth, oral surgery, third molar

## Abstract

**Introduction:** Gemination and fusion are rare developmental anomalies that can present significant diagnostic challenges. Due to the complexity of distinguishing between these conditions, the term “double tooth” is commonly employed in clinical practice. The precise etiology of these anomalies remains uncertain, and their occurrence in permanent dentition—particularly involving molars—is exceptionally rare. This report describes an uncommon case of gemination affecting the mandibular left third molar (tooth 3.8) and provides a comprehensive discussion contextualized within existing literature. The case report was prepared following the CARE guidelines to ensure methodological rigor and completeness.

**Methods:** After an intraoral examination and radiographic assessment—including orthopantomography, periapical radiographs, and cone beam computed tomography (CBCT)—the patient underwent surgical extraction. The procedure involved administering a truncal nerve block to anesthetize the inferior alveolar and lingual nerves, supplemented by local infiltration anesthesia of the buccal nerve. A full-thickness mucoperiosteal flap was elevated, followed by ostectomy and odontotomy to facilitate extraction. The tooth was subsequently removed using a combination of elevators and forceps.

**Results:** Postoperative evaluations conducted at 1.5 and 3 months confirmed complete healing of the surgical site. A detailed analysis of pre- and postoperative radiographic and clinical findings validated the diagnosis of gemination, characterized by coronal continuity with a single root and root canal.

**Conclusions:** Gemination of third molars is exceedingly rare, with only a few cases documented in the literature. To the best of our knowledge, this is the first reported instance of gemination involving the mandibular left third molar (tooth 3.8). This report contributes to the growing body of knowledge on developmental dental anomalies and highlights the importance of thorough differential diagnosis in similar clinical scenarios.

## 1. Introduction

Permanent and deciduous teeth can be affected by developmental anomalies resulting in alterations in the tooth structure. Among the most significant of these anomalies are gemination and fusion, which frequently pose clinical challenges in terms of differential diagnosis, leading to the common use of terms such as “double tooth” [1, 2].

Gemination is a developmental anomaly of tooth shape characterized by the incomplete division of a single tooth germ, resulting in an enlarged tooth with a bifid crown typically accompanied by a single root and root canal [3]. The total number of teeth remains normal when counting the anomalous tooth as one [3]. Clinically, the affected tooth often exhibits a deep groove delineating two distinct crown portions [1, 4]. In contrast, fusion refers to the union of two separate tooth germs at any developmental stage [5]. The extent to which fusion involves dentin, pulp canals, and pulp chambers depends on the developmental stage at which it occurs, resulting in either total or partial fusion [5]. In cases of fusion, the tooth count is one less than normal [5, 6].

Although numerous theories have been proposed regarding the etiology of these anomalies, uncertainty remains [3]. The literature indicates that familial factors play a significant role, alongside environmental influences, trauma, systemic diseases, and associations with conditions such as achondrodysplasia or chondroectodermal dysplasia [1, 3].

Epidemiologically, these anomalies more commonly affect deciduous dentition and are often unilateral. Prevalence rates for unilateral fusion and gemination in deciduous teeth range from 0.5%–1.6% to 2.5% across various studies [2, 7], while prevalence in permanent dentition is approximately 0.08%, varying from 0% to 0.8% [3, 7–9]. A recent study involving 3000 patients reported only one case of gemination (0.03%) and two cases of fusion (0.06%) [1, 10]. Bilateral occurrence rates are significantly lower in both deciduous dentition (0.01%–0.04%) and permanent dentition (0.02%–0.05%), with no discernible gender differences [3, 6]. Both dentitions more frequently exhibit these anomalies in the anterior teeth; cases involving posterior teeth are less common [6].

When anterior teeth are involved, orthodontic or restorative interventions may be necessary to enhance aesthetics [1]. For posterior teeth, there are no established guidelines due to the rarity of such cases; thus, treatment is typically tailored individually [1].

Although gemination and fusion differ conceptually, clinical differentiation between the two anomalies can be challenging, especially when a supernumerary tooth is involved [6]. Nonetheless, Mader's “two-tooth rule” remains a reliable guideline: If the fused tooth is counted as one and the dental arch presents one tooth fewer than normal, it indicates fusion; if the tooth count remains normal, it could be gemination or fusion involving a supernumerary tooth [1, 11].

As previously mentioned, molar gemination cases are exceptionally rare. While some articles have documented upper molar gemination, to our knowledge, very few articles describe gemination involving mandibular third molars [3, 6].

Therefore, this case report aims to present a clinical case of gemination affecting a semi-impacted mandibular left third molar (tooth 3.8).

## 2. Case Report

### 2.1. Diagnosis and Etiology

A 19-year-old female patient presented at the Oral Surgery Division of Fondazione IRCCS Ca' Granda Ospedale Maggiore Policlinico in Milan, reporting severe, persistent pain in the left mandibular region associated with tooth 3.8. Before her visit, an emergency pulpotomy had been performed at a private dental practice. The patient's medical history revealed no known allergies or intolerances to medications or anesthetics; however, she reported moderate mitral valve prolapse and bilateral renal reflux. No previous surgical interventions were documented.

**Figure 1:**
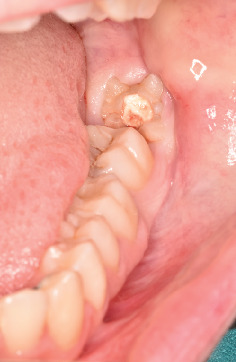
Intraoral objective examination assessment of postpulpotomy element 3.8.

**Figure 2:**
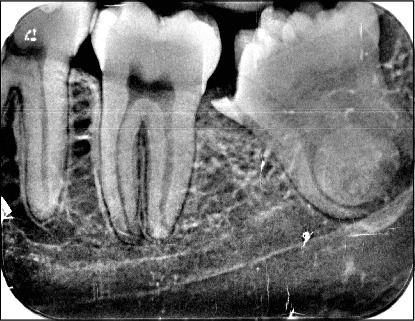
Endoral x-ray shows the absence of enamel and dentin tissue, the relationship between the tooth root and the mandibular canal, and coronal-distal radiolucency.

**Figure 3:**
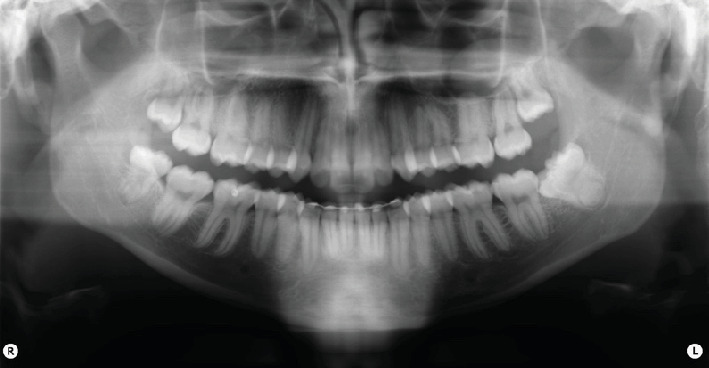
OPG shows altered coronal and root anatomy of element 3.8, deviation of the mandibular canal at the root apex, and a coronal-distal radiolucency.

Intraoral clinical examination revealed an unusual coronal anatomy of tooth 3.8 (Figure 1), prompting further radiographic evaluation. Endoral radiography (Figure 2) and orthopantomography (OPG) (Figure 3), previously obtained at the private dental practice before the pulpotomy, were reviewed to thoroughly assess the anatomical characteristics. Radiographs revealed a distal coronal infection characterized by radiolucency, along with mesial loss of enamel and dentin. Additionally, the OPG indicated mandibular canal deviation at the site of root contact, necessitating cone beam computed tomography (CBCT) to precisely evaluate the relationship between the tooth root and mandibular canal.

**Figure 4:**
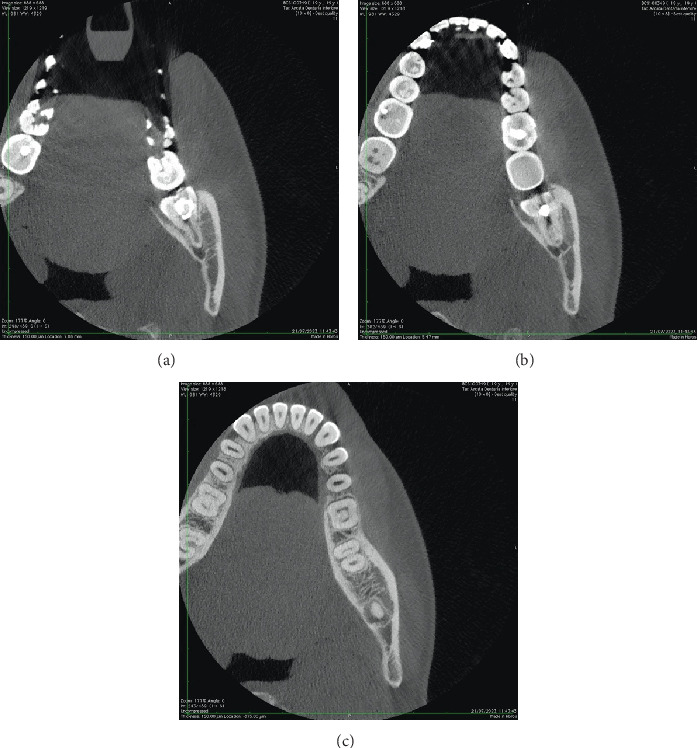
(a–c) Axial CBCT sections of the 3.8 tooth element where the continuity relationship of the root apex with the NVB can be appreciated.

**Figure 5:**
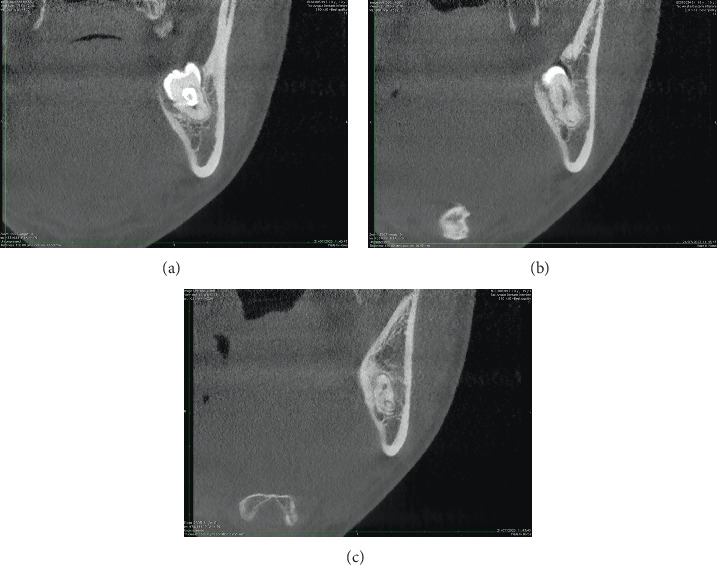
(a–c) Coronal CBCT sections of the 3.8 tooth element illustrate the continuity relationship between the root apex and the NVB.

**Figure 6:**
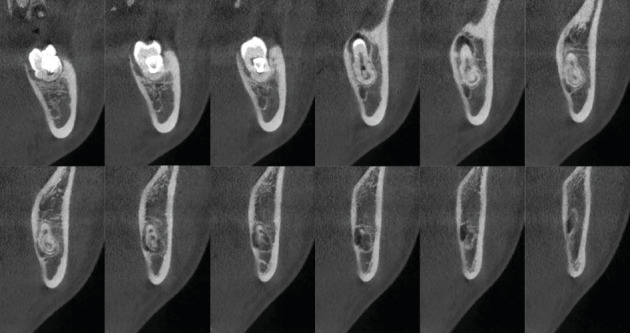
Sagittal CBCT cross-sections of the 3.8 tooth element depict the continuity relationship between the root apex and the NVB.

Axial (Figure 4), coronal (Figure 5), and sagittal CBCT sections (Figure 6) confirmed the absence of the mandibular canal roof at the root apex, indicating communication between the tooth apex and the inferior alveolar neurovascular bundle (NVB).

To alleviate the patient's pain, an emergency pulpotomy was performed, and the tooth was temporarily sealed using glass ionomer cement (GIC) (Ketac Bond Aplicap, 3M ESPE AG, Saint Paul, Minnesota, United States).

The patient reported no history of trauma to the affected area nor previous pathology related to the dental anomaly. She noted the presence of the abnormal tooth since its eruption.

Clinical and radiographic evaluations supported a diagnosis of gemination involving the mandibular left third molar (tooth 3.8). Clinical observation revealed coronal continuity with a distinctive groove oriented in the linguo-vestibular direction, accompanied by a single root and root canal. Confirmation of gemination was further established through direct examination of the tooth cross-section during the surgical procedure [1, 6].

### 2.2. Treatment Objectives

The primary objective of the treatment was the surgical extraction of tooth 3.8 to alleviate pain symptoms and prevent further progression of the carious lesion, which could otherwise lead to pulpal necrosis and an infectious spread to the periapical region, posing a significant risk to the patient.

### 2.3. Treatment Alternatives

An alternative to surgical extraction would have been the retention of the tooth with endodontic treatment. However, this option posed substantial challenges due to the tooth's positioning and the complex anatomy of its pulp chamber and root. Procedures such as shaping, cleansing of canals, and particularly apical obturation would have been considerably difficult or even impracticable.

### 2.4. Treatment Progress

Following written informed consent for both the tooth extraction and the publication of clinical and radiographic data, truncal anesthesia of the inferior alveolar nerve (IAN) and the left lingual nerve was administered using 3% mepivacaine (Molteni Dental s.r.l., Scandicci, FI), supplemented by infiltration anesthesia of the buccal nerve area using 2% mepivacaine with adrenaline (Molteni Dental s.r.l., Scandicci, FI).

**Figure 7:**
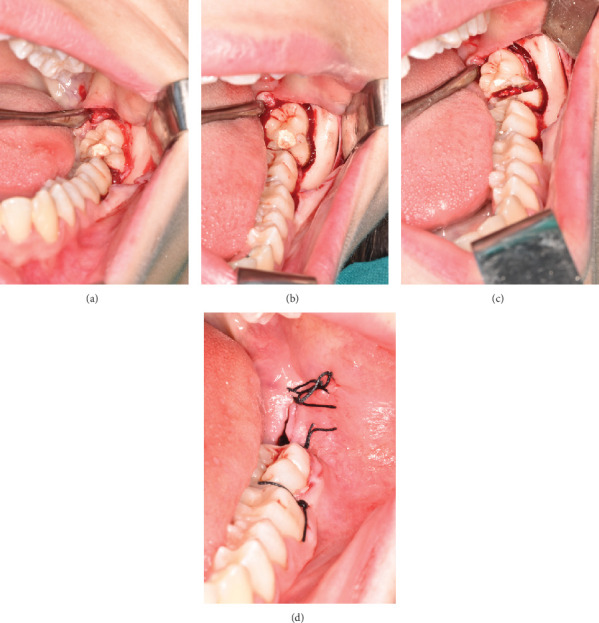
(a) Intraoral photos of the full-thickness mucoperiosteal cleaved surgical flap, (b) vestibulo-distal ostectomy, (c) vestibulo-lingual odontotomy, and (d) postoperative suturing with 3/0 silk.

A full-thickness mucoperiosteal flap was elevated through an intrasulcular incision from tooth 3.6 to tooth 3.8, with an additional distal relieving incision, using a 15C scalpel blade (Henry Schein Inc., Melville, United States). The flap was dissected using a periosteal elevator (Prichard, Hu-Friedy s.r.l., Frankfurt, Germany) and surgical forceps (Hu-Friedy s.r.l., Frankfurt, Germany) (Figure 7a).

To facilitate extraction, vestibular and distal ostectomy was performed using a ball-and-fissure bur mounted on a straight handpiece (Bien Air s.r.l., Milano, Italy) (Figure 7b). Subsequently, vestibulo-lingual odontotomy was performed with the same instrument and finalized manually by inserting a lever between the two tooth segments to achieve fracture (Figure 7c).

The two halves of the tooth were then luxated using a straight elevator and subsequently extracted with forceps. Postextraction, the socket was thoroughly debrided using curettes and surgical spoons and irrigated with saline solution to remove residual fragments and granulation tissue, thereby promoting stable clot formation and optimal healing.

The surgical site was closed using interrupted sutures with 3-0 silk thread (Ethicon INC., Puerto Rico, United States), starting from the distal relieving incision and proceeding to the interproximal areas at the papillary regions of teeth 3.7 and 3.6 (Figure 7d).

Finally, gauze soaked in saline was applied to the surgical area with compression for approximately 5 min to achieve hemostasis [12].

**Figure 8:**
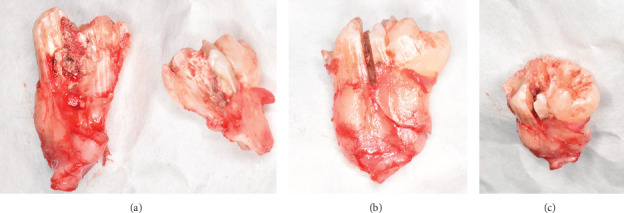
Postoperative photograph (a) of the sectioned 3.8 tooth element and (b, c) of the coronoradicular reconstruction.

Figure 8a illustrates the postoperative appearance of the sectioned tooth 3.8. The coronoradicular reconstruction of the tooth element is depicted in Figure 8b,c.

Figure 8a shows the postoperative photograph of the sectioned 3.8 tooth element. The coronoradicular reconstruction of the element is shown in Figure 8b,c.

### 2.5. Treatment Results

**Figure 9:**
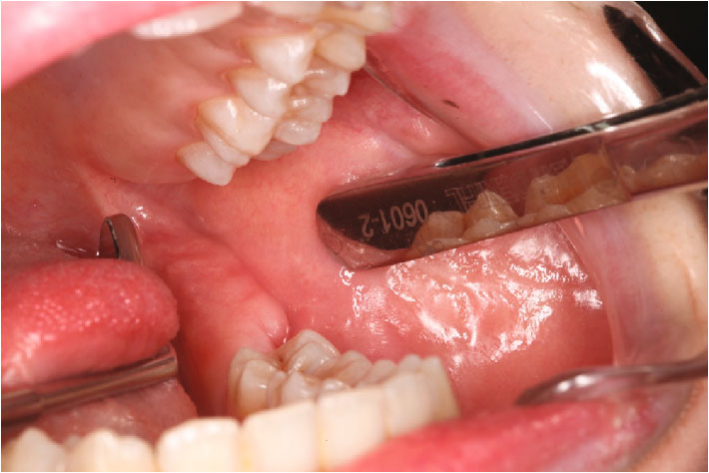
Intraoral photograph 1.5 months after the surgical extraction.

**Figure 10:**
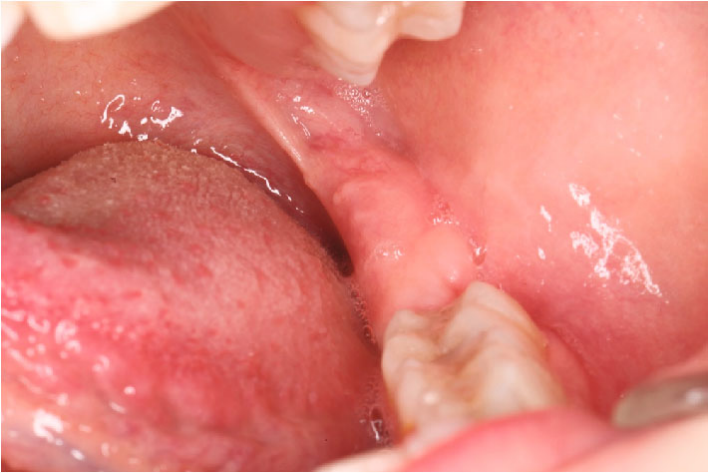
Photograph taken intraorally 3 months postsurgical extraction.

**Figure 11:**
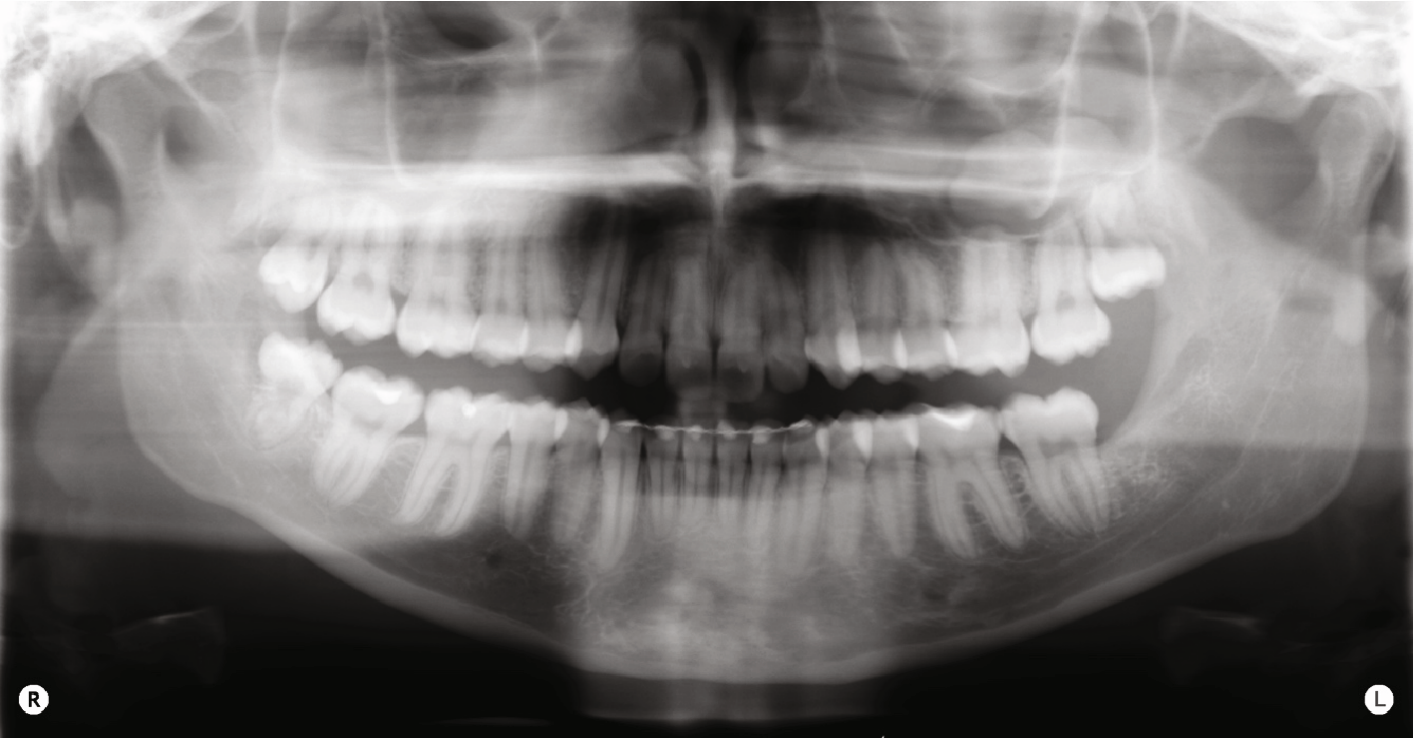
OPG 3 months after surgery shows complete bone healing in zone 3.8, confirmed by the presence of lamellar bone tissue with a high level of ossification.

Sutures were removed, 1 week postoperatively, revealing a wound free of infection. No paresthesia or dysesthesia was reported [13–15]. A subsequent follow-up examination and photographic documentation at 1.5 months demonstrated complete healing of the surgical site and confirmed the continued absence of paresthesia or dysesthesia (Figure 9). At 3 months after extraction, intraoral photography (Figure 10) and OPG (Figure 11) further confirmed complete bone healing at the surgical site.

## 3. Discussion

To date, only five case reports have addressed gemination of mandibular third molars; four reports involve the right lower third molar (tooth 4.8) [1, 6, 16, 17], and only one discusses tooth 3.8 [18]. By reviewing and comparing these five articles, similarities and differences across various aspects can be identified (Table 1).

The publication dates span a considerable range, reflecting long-standing scientific interest in these dental anomalies, with reports ranging from 1985 [16, 17], 2002 [18], to more recent publications in 2020 [6] and 2023 [1].

The reported patient ages vary significantly, indicating that these anomalous teeth do not always cause early clinical issues due to their morphology but may instead become symptomatic later in life due to inflammatory or periodontal conditions. Specifically, Hernandez-Guisado et al. described a 19-year-old patient [18], Sandeep reported a 30-year-old patient [6], Zachariades treated a 45-year-old [16], and Brauer examined a 72-year-old patient [1].

Extraction was consistently the chosen treatment in studies that specified management [6, 18]. The primary indications for tooth extraction were pericoronitis [6, 18] and periodontitis [1]; however, in our case, the primary indication was extensive caries affecting tooth 3.8.

Only our clinical case explicitly reported concurrent medical conditions (moderate mitral valve prolapse and bilateral renal reflux), suggesting no direct association between these conditions and dental gemination.

Regarding language, all articles were published in English [1, 6, 16, 17] except Hernandez-Guisado et al.'s, available in both English and Spanish [18].

Notably, Hernandez-Guisado et al.'s article [18], the sole report concerning tooth 3.8, described the anomaly as gemination; however, it is more plausible that this was a fusion between tooth 3.8 and a supernumerary tooth. Differential diagnosis typically relies on tooth counting according to Mader's rule [1, 11]. However, this method is less reliable when a supernumerary tooth is involved, as in the case described in the article by Hernandez-Guisado, complicating diagnosis [1, 11].

Several studies highlight additional criteria for differential diagnosis beyond tooth counting, notably external and endodontic anatomy [19, 20]. According to Song et al. [19], geminated teeth typically possess a single pulp canal and one root, while fused teeth exhibit separate pulp canals combined by dentin.

Hernandez-Guisado et al. further clarified that gemination generally involves one root and two crowns, whereas fusion has two crowns and one or two roots with separate canals [18]. In the case reported by Hernandez-Guisado et al., the extracted tooth displayed two distinct crowns and separate endodontic anatomy. Specifically, it featured three roots: two distinct ones and a third resulting from fusion between the distal root of tooth 3.8 and the mesial root of tooth 3.9, each containing separate canals.

Sandeep et al. [6] also noted that fusion typically shows two distinct pulp chambers radiographically, whereas gemination displays a single pulp chamber. Hernandez-Guisado et al.'s radiological findings indicated separate pulp chambers, supporting the diagnosis of fusion [18]. Thus, the described tooth appears to represent fusion between teeth 3.8 and 3.9 at the middle third, distinct at the coronal third, featuring three roots, one resulting from fused roots [18].

Fusion, unlike gemination, involves the union of two distinct dental elements and may result in the presence of supernumerary teeth [21].

The origin of supernumerary teeth is still debated. Several theories have been proposed, including the “dental germ dichotomy” theory and the “hyperactivity theory,” which attributes these teeth to abnormal and independent activity of the dental lamina [22]. Genetic factors also play a crucial role, with familial recurrence suggesting complex hereditary patterns, such as autosomal dominant traits with variable penetrance and possible sex-linked mechanisms [23].

Supernumerary teeth are often associated with developmental syndromes, like cleft lip and palate, cleidocranial dysostosis, and Gardner syndrome, as well as rarer conditions like Fabry–Anderson syndrome and incontinentia pigmenti [24].

Prevalence in the general population ranges from 0.1% to 3.8% in permanent dentition and from 0.3% to 0.8% in primary dentition, with higher rates reported among East Asian individuals and in males compared to females [24].

Supernumerary teeth are typically classified according to their morphology and position [24, 25]. Tuberculate supernumeraries are generally larger, often barrel-shaped, and characterized by multiple cusps; they tend not to erupt completely. Supplemental teeth resemble normal teeth and are more commonly observed in the primary dentition, with the maxillary lateral incisor being the most frequent example. Another form is represented by odontomas, which are hamartomatous malformations subdivided into two types: complex composite odontomas, consisting of a disorganized mass of dental tissues without a defined anatomical structure, and compound composite odontomas, which display some morphological similarity to normal teeth despite their structural abnormalities [24, 25].

In terms of their position, supernumerary teeth can be classified into several types [25]. Mesiodens are located between the maxillary central incisors and may be either erupted or impacted. Paramolars are small additional teeth situated buccally or lingually to the maxillary molars, while distomolars are found distal to the third molars.

The mesiodens is the most common type, accounting for about 60% of all supernumerary teeth [26]. While supernumerary teeth generally occur singly, multiple occurrences are rare and often linked to syndromic conditions [24].

It is crucial to note that supernumerary teeth are exclusively associated with fusion cases. Gemination, as previously explained, involves the splitting of a single dental germ and does not lead to the formation of supernumerary teeth.

Therefore, no authentic case of gemination affecting tooth 3.8 has been documented in the literature, since Hernandez-Guisado et al.'s report appears to represent fusion rather than gemination.

To enhance accuracy, transparency, and utility, this case report adheres to the CAse REport (CARE) checklist [27], detailed in Table S1 (Supporting Information).

## 4. Conclusion

The differential diagnosis between fusion and gemination is frequently challenging, particularly when supernumerary teeth are involved. Despite this complexity, surgical extraction remains the most recommended treatment for gemination and fusion of third molars.

This case report contributes significantly to the existing literature by presenting a documented case of gemination involving tooth 3.8, a condition for which no previously published cases exist.

## Figures and Tables

**Table 1:** Summary of the main features of published studies to date reporting mandibular third molars with gemination.

**Authors and year of publication**	**Publication language**	**Patient's age**	**Patient-associated diseases**	**Tooth element with gemination**	**Tooth avulsion indications**	**Surgical technique adopted for tooth avulsion**
Brauer H.U. et al., 2023 [1]	English	72 years old	N.R.	4.8	4.8 distal periodontal pocket	N.R.
Sandeep S. et al., 2020 [6]	English	30 years old	N.R.	4.8	Pericoronitis	Surgical extraction with local anesthesia.
Zachariades N. et al., 1985 [16]	English	45 years old	N.R.	4.8	N.R:	N.R.
Ruprecht A. et al., 1985 [17]	English	N.R.	N.R.	4.8	N.R.	N.R.
Hernandez-Guisado et al., 2002 [18]	Spanish and English	19 years old	N.R.	3.8	Pericoronitis	Extraction with osteotomy using a bayonet incision.

Abbreviation: N.R., not reported.

## Data Availability

The authors confirm that the data supporting the findings of this study are available within the article.
