# Supplementary material for: Clinical and Radiographic Features of Mandibular Third Molar Gemination: A Case Report and Literature Review
Source: Case Rep Dent. 2025 May 20;2025:8934034. doi: 10.1155/crid/8934034 (PMC12116212; doi:10.1155/crid/8934034)
Supplement: Supporting Information — Additional supporting information can be found online in the Supporting Information section. Table S1 CARE Checklist of information to include when writing a case report. [file 8934034.f1.docx]

**Supplementary Materials**

**Table S1:** CARE Checklist of information to include when writing a case report.

| **Topic** | **Item** | **Checklist item description** | **Reported on Line** |
| --- | --- | --- | --- |
| **Title** | 1 | The diagnosis or intervention of primary focus followed by the words “case report” | 1 |
| **Keywords** | 2 | 2 to 5 keywords that identify diagnoses or interventions in this case report, including "case report" | 40 |
| **Abstract (no references)** | 3a | Introduction: What is unique about this case and what does it add to the scientific literature? | 82-84 |
|  | 3b | Main symptoms and/or important clinical findings | 9 and 109 |
|  | 3c | The main diagnoses, therapeutic interventions, and outcomes | 111-114 and 169-172 |
| **Introduction** | 4 | One or two paragraphs summarizing why this case is unique (may include references) | 82-86 and 283-284 |
| **Patient Information** | 5a | De-identified patient-specific information | / |
|  | 5b | Primary concerns and symptoms of the patient | 90-91 |
|  | 5c | Medical, family, and psycho-social history including relevant genetic information | 92-94 |
| **Clinical Findings** | 6 | Describe significant physical examination (PE) and important clinical findings | 95 and 112-114 |
| **Timeline** | 7 | Historical and current information from this episode of care organized as a timeline | / |
| **Diagnostic Assessment** | 8a | Diagnostic testing (such as PE, laboratory testing, imaging, surveys) | 96-104 |
|  | 8b | Diagnostic challenges (such as access to testing, financial, or cultural) | / |
|  | 8c | Diagnosis (including other diagnoses considered) | 71-76 and 110-114 |
|  | 8d | Prognosis (such as staging in oncology) where applicable | / |
| **Therapeutic Intervention** | 9a | Types of therapeutic intervention (such as pharmacologic, surgical, preventive, self-care) | 169-172 |
|  | 9b | Administration of therapeutic intervention (such as dosage, strength, duration) | / |
|  | 9c | Changes in therapeutic intervention (with rationale) | / |
| **Follow-up and Outcomes** | 10a | Clinician and patient-assessed outcomes (if available) | / |
|  | 10b | Important follow-up diagnostic and other test results | 215-220 |
|  | 10c | Intervention adherence and tolerability (How was this assessed?) | / |
|  | 10d | Adverse and unanticipated events | / |
| **Discussion** | 11a | A scientific discussion of the strengths AND limitations associated with this case report | 297-298 |
|  | 11b | Discussion of the relevant medical literature with references | 239-284 |
|  | 11c | The scientific rationale for any conclusions (including assessment of possible causes) | / |
|  | 11d | The primary “take-away” lessons of this case report (without references) in a one paragraph conclusion | 294-298 |
| **Patient Perspective** | 12 | The patient should share their perspective in one to two paragraphs on the treatment(s) they received | / |
| **Informed Consent** | 13 | Did the patient give informed consent? Please provide if requested | Yes |
